# Predictors of diagnosis and survival in idiopathic pulmonary fibrosis and connective tissue disease-related usual interstitial pneumonia

**DOI:** 10.1186/s12931-014-0154-6

**Published:** 2014-12-04

**Authors:** Teng Moua, Ana C Zamora Martinez, Misbah Baqir, Robert Vassallo, Andrew H Limper, Jay H Ryu

**Affiliations:** Division of Pulmonary and Critical Care Medicine, Mayo Clinic, 200 First St. SW, Rochester, MN 55901 USA

**Keywords:** Idiopathic pulmonary fibrosis, Usual interstitial pneumonia, Connective-tissue disease interstitial lung disease

## Abstract

**Background:**

Although usual interstitial pneumonia (UIP) appears to portend better survival when associated with connective tissue disease (CTD-UIP), little is known about the presenting clinical, radiologic, and pathologic features that differentiate pathologically confirmed UIP with CTD from idiopathic pulmonary fibrosis (IPF). In patients with atypical radiologic and clinical features, what specific findings predict underlying IPF vs. CTD-UIP diagnosis and their respective long term survival?

**Methods:**

A large retrospective cohort analysis was done of consecutive patients seen from 1995 through 2010 with biopsy confirmed UIP completed or reviewed at our institution. CTD-UIP was defined by independent rheumatology consultation with exclusion of all other secondary causes of lung fibrosis. Primary clinical data was collected and compared for IPF and CTD-UIP along with logistic regression performed for predictors of disease likelihood and Cox proportional hazards analysis for predictors of survival.

**Results:**

Six hundred and twenty five patients were included in the study of which 89 had diagnosed CTD-UIP representing 7 disease entities. Survival was better among those with CTD-UIP except in UIP associated with rheumatoid arthritis, which had similar presenting features and survival to IPF. Predictors of underlying CTD included female gender, younger age, positive autoimmune serology, and inconsistent presenting radiologic findings. Only age and forced vital capacity corrected for a priori covariates were predictive of survival in CTD-UIP.

**Conclusions:**

UIP pathology occurs frequently among patients with atypically presenting clinical and radiologic features, and may represent IPF or CTD-UIP with improved prognosis if underlying CTD is diagnosed. Presenting radiologic and pathologic features alone are not predictive of underlying secondary cause or survival between the two groups.

## Introduction

Usual interstitial pneumonia (UIP) is characterized by temporally heterogenous parenchymal fibrosis with architectural distortion, interstitial thickening, fibroblast foci, and honeycombing [1]. Although a defining pathologic finding in idiopathic pulmonary fibrosis (IPF), it has been found in other chronic fibrotic lung disease such as the connective tissue-disease associated interstitial lung disease (CTD-ILD) [2,3], chronic hypersensitivity pneumonitis (HP) [4], sarcoidosis [5], and advanced asbestosis [6].

Current classification of the idiopathic interstitial pneumonias (IIP) allows not only pathological distinction of fibrotic disease, but implied characteristic clinical and prognostic significance [7]. For example, it is well known that UIP has worse prognosis than non-specific interstitial pneumonia (NSIP), the two most commonly presenting pathologies [8,9]. Both again may be idiopathic or associated with known etiologies, which has clinical significance in terms of survival and response to therapy [10]. Prior studies have suggested secondary UIP such as that seen in certain connective-tissue diseases (CTD-UIP) may have better prognosis and survival than IPF [2,10]. Other studies have been conflicting regarding better survival in difficult to diagnose CTD or all CTD-ILD [11,12]. Specific features of disease severity such as presenting CT findings [13] (reticulation vs. presence of honeycombing), pulmonary function testing [14,15], and physiology scores [16,17] have been used to predict disease progression and mortality.

Surgical lung biopsy is often avoided in those with typical radiologic features consistent with IPF or clinical association with connective-tissue disease. Even so, many biopsies are obtained because of atypically presenting radiologic or clinical features that do not allow for directed management or discussion of a care plan. As survival in CTD-UIP has been accepted as better than IPF, we sought to review the clinical, radiologic, and pathologic features of all biopsy proven UIP patients with either IPF or CTD seen at our institution, assessing for all-cause mortality. We hypothesized that variations in presenting clinical, pathologic, and radiologic findings may differentiate IPF from CTD-UIP in terms of predicting diagnosis and survival.

## Materials and methods

Institutional IRB approval was obtained (IRB# 11–003506). A computer-assisted search of the pathological database was performed and consecutive patients with biopsy proven UIP seen at Mayo Clinic Rochester from 1995–2010 were included in the initial review. Biopsies were obtained either at Mayo Clinic Rochester or outside institutions, with pathological UIP defined using standard criteria [18] by experienced pulmonary pathologists at the time of clinical assessment.

Pathology from outside biopsies was re-reviewed at our institution at the time of referral. Atypical pathological findings in addition to underlying UIP if noted by the reading pathologist were collated and included presence but not predominance of any of the following: 1) poorly formed granuloma, 2) organizing pneumonia, 3) lymphoid aggregates or hyperplasia, 4) chronic inflammation, and 5) diffuse alveolar damage (DAD). Patients were excluded if pathologic findings only suggested possible UIP or had UIP-like features but were not consistent with UIP criteria.

IPF was diagnosed according to recent consensus guideline as biopsy-confirmed UIP without clinical evidence of a known secondary etiology [18]. In our cohort, all patients presenting with signs or symptoms suggestive of rheumatologic disease underwent directed autoimmune serologic testing, and if positive were considered undifferentiated connective tissue disease (UCTD) if no further definable rheumatologic disease was found. For patients with positive screening serology but no rheumatologic symptoms, IPF was still considered the underlying diagnosis. If initial CT was atypical for IPF but progressed over time to become probable or consistent UIP along with worsened respiratory symptoms, IPF was also considered the final diagnosis. Underlying CTD was defined by standard criteria through formal Rheumatology consultation at the time of referral or if diagnosed previously. These included rheumatoid arthritis (RA), scleroderma (Scl), dermatomyositis/polymyositis (DM/PM), mixed-connective tissue disease (MCTD), lupus erythematosus (SLE), primary Sjögren’s syndrome, and undifferentiated connective tissue disease (UCTD). As there is variability in the literature regarding UCTD definitions, we applied the broad definition as outlined by Kinder et al. [19] in conjunction with Rheumatology consultation. Patients with UIP associated with hypersensitivity pneumonitis, sarcoidosis, or post-inflammatory injury (radiation or drug toxicity) were excluded from the analysis.

Manually collected baseline clinical data included age at time of biopsy, gender, patient reported duration of respiratory symptoms prior to clinical assessment, smoker status and pack years, history of gastroesophageal reflux disease (GERD), and treatment with any immunosuppressant or anti-inflammatory regimen defined as directed or empiric use at a high or therapeutic level greater than four weeks. Clinical follow-up was through date of last visit at our institution, with date of death reviewed in the medical record or by US Social Security Death Index (US SSDI) search (search date 12/10/2013). Transplant free survival and all-cause mortality were used as parameters.

Screening circulating autoimmune serologies if performed by the evaluating clinician were reviewed and collated, defined by positive laboratory findings in any of the following frequently obtained serological studies: ANA antibody titer and serology, antibody to extractable nuclear antigen (ENA), rheumatoid factor (RF), anti-CCP, SS-A or La, SS-B or rho, anti-Jo-1 synthetase, anti-RNP, anti-Scl-70, and anti-Smith antibody. Decision to obtain screening serology studies was clinician dependent based on presentation at the time of referral.

Pulmonary function data was reviewed for baseline presenting percent predicted (% pred) pre-bronchodilator forced vital capacity (FVC), forced expiratory volume in one second (FEV_1_), total lung capacity (TLC), and diffusing capacity for carbon monoxide (DLCO). Earliest presenting PFT findings at the time of lung fibrosis evaluation either at our institution or the referring institution were analyzed if multiple studies were available.

Presenting chest tomography (CT) studies obtained closest to the date of clinical assessment for lung fibrosis were reviewed by one of the authors (TM) and compared to initial Mayo reading radiologist interpretation. Classification into one of three categories was done based on recent IPF guideline [18]: 1) consistent with UIP (bibasilar reticular, honeycombing, peripheral distribution, absence of other features, 2) possible UIP (peripheral reticular, bibasilar, absence of other features), and 3) probable NSIP or inconsistent with UIP (subpleural ground glass or mild reticulation, upper lobe distribution as well as bibasilar, other features of atypical scarring or fibrosis).

Statistical analysis was performed on JMP Software (Vers 10.0, Cary NC). Comparative analysis was done using Fisher’s exact, Chi-Square, or T-test. Univariate and multivariate logistic regression was performed adjusting for a priori covariates of age, gender, smoker status, and baseline percent predicted FVC and DLCO. Survival analysis for all-cause mortality was done using the Kaplan Meier method with Log rank. Cox proportional hazards analysis adjusted and unadjusted was done using the same a priori covariates above to delineate predictors of death. Missing data was reviewed and where considered random, was adjusted using complete case analysis (list-wise deletion). Subjects were censored if date of death could not be obtained because of foreign status, underwent transplantation, or were suspected alive at the time of US SSDI search (12/10/2013). P values <0.05 were considered statistically significant.

## Results

Six hundred and sixty eight consecutive patients with UIP confirmed biopsies were reviewed for the study. Forty three patients had UIP associated with other etiologies (chronic HP, drug toxicity, sarcoidosis, and asbestosis) and were excluded. Eighty nine were diagnosed with eventual CTD after formal Rheumatology consultation and included in the study, of which the majority (84%) had lung biopsy prior to diagnosis. The remaining 536 were considered IPF. Baseline demographics and underlying CTD diagnoses associated with UIP are presented in Tables 1 and 2. CTD-UIP patients were younger at the time of biopsy (59.3 vs. 64.7 yrs (P <0.0001), predominantly female (58% vs. 36%, P <0.0001) with less smoking history (48% ever smokers vs. 62%, P =0.003), and had higher prevalence of circulating autoimmune antibodies compared to IPF at the time of presentation (78% vs. 29%, P <0.001). Duration of patient reported respiratory symptoms and distribution of presenting PFT and HRCT patterns were no different between the two groups, though CTD-UIP patients were more likely to receive treatment (91% vs 67%; P <0.0001). Atypical biopsy findings were also more frequent in CTD-UIP compared to IPF (34% vs 15%, P <0.0001) consisting predominantly of chronic inflammation and lymphoid hyperplasia. UIP with poorly formed granulomas, chronic inflammation, and organizing pneumonia were predominant atypical pathologic findings seen in IPF. The majority of CTD diagnoses associated with UIP were UCTD (34%) and RA (27%).

**Table 1:** **Baseline demographics**

**Characteristic**	**Total (N = 625)**	**IPF (N = 536)**	**CTD-UIP (N = 89)**	**P** ^**¶**^
Age at biopsy, Yrs	63.9 ± 9.9	64.7 ± 9.4	59.3 ± 11.5	**<0.0001**
Gender, M/F (%)	382/243 (61/39)	345/191 (64/36)	37/52 (42/58)	**<0.0001**
Smoking history, N (%)				**0.003**
Never	250 (40)	204 (38)	46 (52)	
Former	364 (58)	325 (60)	39 (44)	
Current	11 (2)	7 (2)	4 (4)	
Reported symptom duration prior to ILD diagnosis, months (range)	23.9 ± 25.3 (1–180)	23.6 ± 24.7 (0–180)	25.3 ± 28.6 (0–120)	0.56
Clinical GERD, N (%)	362 (58)	312 (58)	50 (56)	0.72
Autoimmune serology, N (%)	180 (39%); N = 460 tested	111 (29%); N = 378 tested	69 (78%); N = 82 tested	**<0.001**
FEV_1_, % pred, mean ± SD	68 ± 17; N = 552	68.4 ± 16.8; N = 475	65.7 ± 18.2; N = 77	0.21
FVC, % pred, mean ± SD	64.6 ± 17.7; N = 557	64.6 ± 17.3; N = 481	64 ± 19.7; N = 76	0.76
TLC, % pred, mean ± SD	67.2 ± 14.5; N = 478	67.2 ± 14.2; N = 412	67.7 ± 16.8; N = 66	0.77
DLCO, % pred, mean ± SD	47.5 ± 15.6; N = 508	47.6 ± 15.7; N = 438	47 ± 15.4; N = 70	0.74
HRCT at Bx, N (%)	N = 584	N = 502	N = 82	0.09
Consistent with UIP	136 (23)	119 (24)	17 (21)	
Possible UIP	266 (46)	235 (47)	31 (38)	
NSIP or inconsistent UIP	182 (31)	148 (29)	34 (41)	
Atypical pathology findings, N (%)	113 (18)	83 (15)	30 (34)	**<0.0001**
Treated, N (%)	438 (70)	357 (67)	81 (91)	**<0.0001**
Lung transplant, N (%)	33 (5)	27 (5)	6 (7)	0.51
Deaths, N (%)	442 (71)	391 (73)	51 (57)	**0.003**
Median follow-up in months, median (range)	49.5 (1–223)	47.7 (1–198)	79.4 (1–223)	-

All data presented as Mean ± SD unless otherwise noted.

^¶^P value is for IPF vs. CTD-UIP.

GERD = gastroesophageal reflux disease, ILD = interstitial lung disease, NSIP = nonspecific interstitial pneumonia, UIP = usual interstitial pneumonia.

**Table 2:** **Specific connective-tissue disease distributions**

**CTD subtype (N = 89)**	**N (%)**
RA	24 (27)
SLE	2 (2)
Scleroderma (systemic sclerosis)	13 (15)
MCTD	2 (2)
DM/PM	13 (15)
Sjogren’s syndrome	5 (5)
Undifferentiated CTD	30 (34)

CTD = connective tissue disease, DM/PM = dermatomyositis/polymyositis, MCTD = mixed connective tissue disease, RA = rheumatoid arthritis, SLE = systemic lupus erythematosus.

Among patients with pathologically confirmed UIP, younger age, female gender, positive autoimmune serology, and non-UIP consistent radiologic patterns unadjusted by logistic regression were predictive of CTD diagnosis (Table 3). After adjusting for a priori covariates (age, gender, smoking history, percent predicted FVC and DLCO), age, positive autoimmune serology, and inconsistent CT findings remained predictive of underlying CTD.

**Table 3:** **Clinical predictors of IPF vs. CTD-UIP diagnosis**

	**Univariable OR (95% CI)**	**P value**	**Multivariable OR (95% CI)**	**P value**
Age at biopsy	1.06 (1.03-1.09)	**<0.0001**	1.06 (1.02-1.08)	**0.0012**
Gender (male risk)	2.52 (1.44-4.49)	**<0.0001**	1.87 (0.95-3.7)	0.07
Duration of symptoms prior to biopsy	1.01 (0.99-1.02)	0.27	1.01 (0.99-1.03)	0.19
Positive autoimmune serology	0.08 (0.04-0.15)	**<0.0001**	0.09 (0.04-0.17)	**<0.0001**
FEV_1,_ % pred	1.01 (0.99-1.03)	0.17	1.03 (0.97-1.10)	0.27
FVC, % pred	1.00 (0.98-1.02)	0.61	0.99 (0.92-1.07)	0.73
DLCO, % pred	1.01 (0.99-1.03)	0.31	1.02 (0.99-1.05)	0.15
TLC, % pred	0.99 (0.98-1.02)	0.74	0.98 (0.94-1.03)	0.36
Initial HRCT pattern				
Probable UIP	1.00 (0.42-2.27)	0.98	0.79 (0.29-2.05)	0.64
Probable NSIP or inconsistent with UIP	0.40 (0.17-0.86)	**0.02**	0.31 (0.11-0.76)	**0.01**
Atypical findings on biopsy	0.36 (0.19-0.69)	**0.003**	0.56 (0.27-1.22)	0.56

% pred = percent predicted.

Complete case analysis.

Table 4 represents univariable and multivariable adjusted predictors of survival among all UIP patients. Initial unadjusted analysis suggested both smoking (HR 1.32 [1.04-1.69], P =0.023) and positive autoimmune serology were predictive of survival (HR 0.72, [0.55-0.93], P = 0.02), though were no longer predictive after correction for age, gender, FVC, and DLCO.

**Table 4:** **Clinical predictors of death in all UIP pathology**

	**Univariable HR (95% CI)**	**P value**	**Multivariable, HR (95% CI)**	**P value**
Age at biopsy	1.04 (1.02-1.05)	**<0.0001**	1.03 (1.02-1.05)	**<0.0001**
Male gender	1.64 (1.29-2.11)	**<0.001**	1.55 (1.19-2.03)	**0.0013**
Smoking Hx	1.32 (1.04-1.69)	**0.023**	1.14 (0.88-1.49)	0.32
Duration of symptoms prior to biopsy	1.00 (0.99-1.01)	0.89	0.99 (0.99-1.00)	0.75
Positive autoimmune serology	0.72 (0.55-0.93)	**0.02**	0.83 (0.63-1.08)	0.18
GERD	0.96 (0.76-1.22)	0.75	0.94 (0.74-1.20)	0.64
FEV_1,_ % pred	0.98 (0.98-0.99)	**<0.0001**	0.99 (0.97-1.02)	0.64
FVC, % pred	0.98 (0.97-0.99)	**<0.0001**	0.99 (0.98-0.99)	**0.003**
DLCO, % pred	0.98 (0.98-0.99)	**<0.0001**	0.99 (0.98-0.99)	**0.017**
TLC, % pred	0.98 (0.97-0.99)	**<0.0001**	0.99 (0.98-1.02)	0.78
Initial HRCT pattern				
Probable UIP	0.80 (0.59-1.09)	0.15	0.83 (0.61-1.13)	0.23
Probable NSIP or inconsistent with UIP	0.88 (0.64-1.22)	0.88	0.96 (0.70-1.33)	0.79
Atypical findings on biopsy	0.78 (0.56-1.07)	0.13	0.83 (0.59-1.14)	0.27

% pred = percent predicted.

Complete case analysis.

Multivariable adjustment for age, gender, smoking hx, FVC, and DLCO.

GERD = gastroesophageal reflux disease, NSIP = nonspecific interstitial pneumonia, UIP = usual interstitial pneumonia.

Given the current UIP cohort was predominantly representative of IPF patients, subgroup analysis was performed on CTD-UIP and IPF separately for clinical predictors of survival (Tables 5 and 6). While age, gender, FVC, and DLCO adjusted for a priori covariates were predictive of survival in IPF patients, only age and FVC adjusted were predictive in CTD-UIP. Initial univariable analysis suggested smoking history, FEV1, and FVC were predictive in CTD but were no longer after correction for age and gender. Despite female gender being predictive of CTD diagnosis, gender was not predictive of survival among CTD patients while in IPF male gender portended worse survival in both univariable and multivariable analysis.

**Table 5:** **Subgroup analysis of clinical predictors for death in CTD-UIP**

	**Univariable HR (95% CI)**	**P value**	**Multivariable, HR (95% CI)**	**P value**
Age at biopsy	1.06 (1.02-1.11)	**0.0031**	1.05 (1.01-1.11)	**0.03**
Gender (male risk)	1.91 (0.83-4.34)	0.13	1.05 (0.39-2.80)	0.92
Smoking Hx	4.24 (1.76-11.80)	**0.0010**	2.74 (0.98-9.01)	0.06
Duration of symptoms prior to biopsy	1.00 (0.99-1.02)	0.69	1.01 (0.98-1.03)	0.60
GERD	0.71 (0.31-1.60)	0.41	0.64 (0.25-1.60)	0.33
FEV_1,_ % pred	0.97 (0.95-0.99)	**0.04**	0.99 (0.90-1.13)	0.97
FVC, % pred	0.98 (0.96-0.99)	**0.02**	0.95 (0.92-0.98)	**0.01**
DLCO, % pred	0.95 (0.92-0.99)	**0.005**	0.99 (0.95-1.03)	0.58
TLC, % pred	0.96 (0.93-0.99)	**0.01**	0.99 (0.92-1.07)	0.88
Initial HRCT pattern				
Probable UIP	1.78 (0.46-11.67)	0.44	2.06 (0.48-14.31)	0.35
Probable NSIP or inconsistent with UIP	2.34 (0.64-14.99)	0.22	2.83 (0.71-19.10)	0.15
Atypical findings on biopsy	0.39 (0.11-1.02)	0.06	0.68 (0.19-1.91)	0.49

% pred = percent predicted.

Complete case analysis.

Multivariable adjusting for age, gender, smoking hx, FVC, and DLCO.

**Table 6:** **Subgroup analysis of clinical predictors of death in IPF**

	**Univariable HR (95% CI)**	**P value**	**Multivariable, HR (95% CI)**	**P value**
Age at biopsy	1.03 (1.02-1.04)	**<0.0001**	1.03 (1.01-1.04)	**0.0002**
Gender (male risk)	1.48 (1.15-1.93)	**0.003**	1.52 (1.15-2.02)	**0.003**
Smoking Hx	1.13 (0.88-1.46)	0.34	1.04 (0.77-1.37)	0.79
Duration of symptoms prior to biopsy	0.99 (0.99-1.00)	0.51	0.99 (0.99-1.00)	0.40
Autoimmune serology	0.89 (0.65-1.19)	0.43	0.95 (0.69-1.27)	0.72
GERD	1.05 (0.82-1.35)	0.66	1.02 (0.80-1.31)	0.87
FEV_1,_ % pred	0.98 (0.98-0.99)	**0.0004**	0.99 (0.97-1.02)	0.53
FVC, % pred	0.98 (0.98-0.99)	**0.0001**	0.99 (0.98-0.99)	**0.03**
DLCO, % pred	0.99 (0.98-0.99)	**0.0001**	0.99 (0.98-0.99)	**0.02**
TLC, % pred	0.98 (0.98-0.99)	**0.0012**	1.00 (0.99-1.02)	0.65
Initial HRCT pattern				
Probable UIP	0.76 (0.56-1.04)	0.09	0.78 (0.57-1.08)	0.13
Probable NSIP or inconsistent with UIP	0.91 (0.65-1.27)	0.56	0.96 (0.69-1.35)	0.82
Atypical findings on biopsy	0.99 (0.70-1.39)	0.98	0.96 (0.67-1.35)	0.82

% pred = percent predicted.

Complete case analysis (list-wise deletion).

Multivariable adjusting for age, gender, smoking hx, FVC, and DLCO.

**Figure 1:**
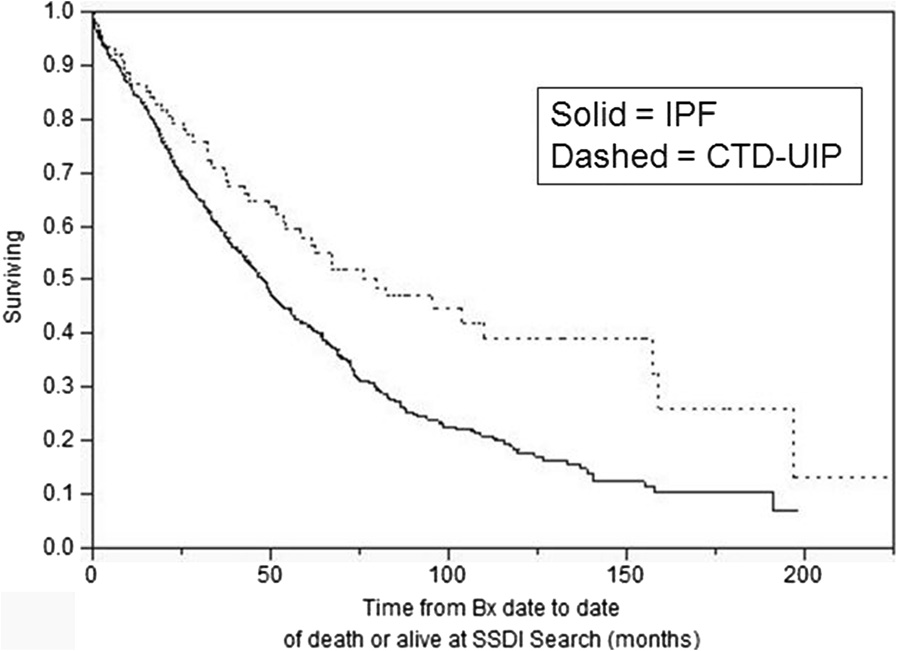
**Survival in IPF vs. CTD-UIP; (P = 0.0005 Log-rank).**

Figure 1 compares transplant-free survival from biopsy to date of death from any cause (all-cause mortality) between IPF and CTD-UIP. Survival among CTD-UIP was notably better (median 79.4 vs. 47.7 months, Log rank P = 0.0005).

**Figure 2:**
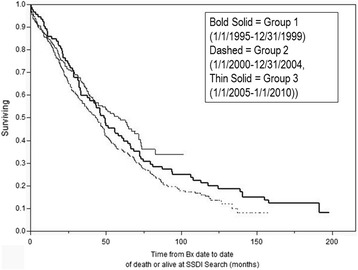
**Survival in IPF stratified by 5 year periods (1995–2010); (P = 0.03 Log Rank).**

Figure 2 depicts survival in biopsy confirmed IPF patients stratified by 5 year intervals (a) 1995–1999, b) 2000–2004, c) 2005–2010). Survival appeared greatest in patients biopsied from 2005–2010 (median 58.3 (42.3-71.5) months) and least in those biopsied between 2000–2004 (median 43.7 (34.2-48.9); (Log rank P = 0.03)).

**Figure 3:**
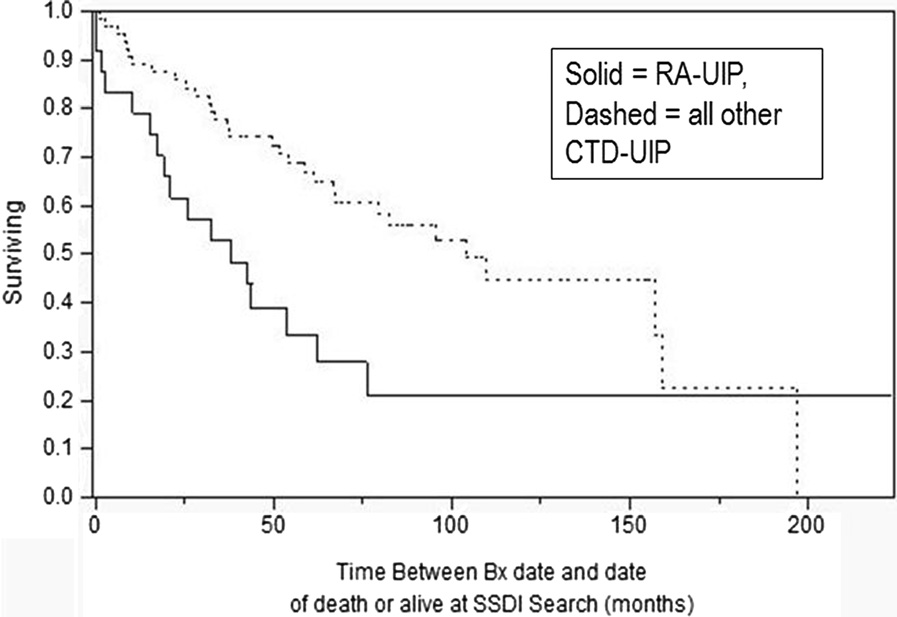
**Survival in RA-UIP vs. all other CTD-UIP; (P = 0.0163 Log Rank).**

**Figure 4:**
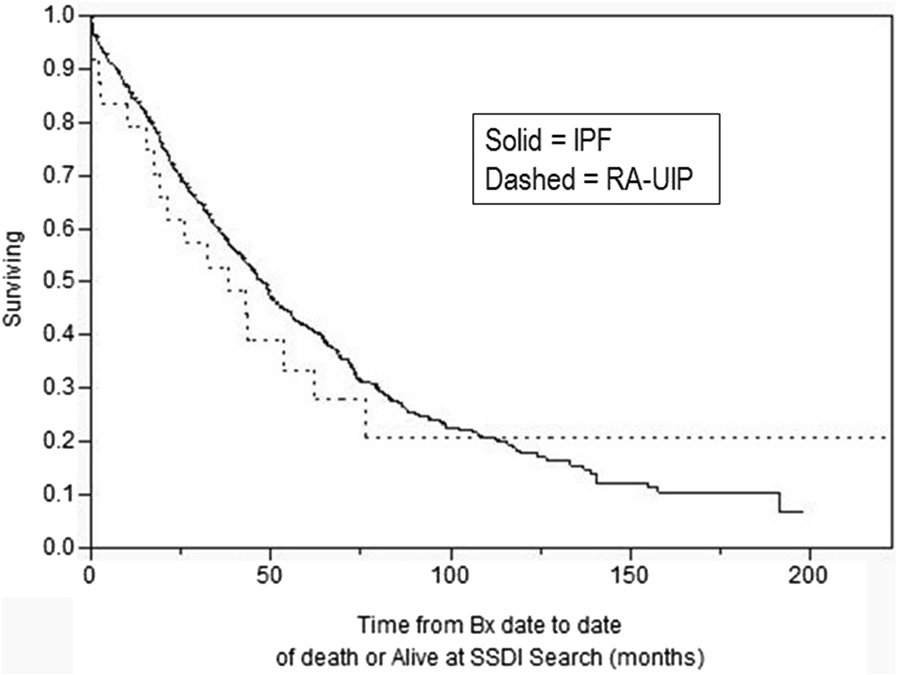
**Survival in RA-UIP vs. IPF; (P =0.76 Log Rank).**

As CTD-UIP represents a heterogenous group of underlying diseases, and there have been recent reports of rheumatoid related lung fibrosis representing more aggressive disease [20,21], subgroup analysis of survival among RA-UIP vs all other CTD-UIP and IPF was performed and depicted in Figures 3 and 4. RA-UIP appears to have worse survival compared to the other CTDs (median 38 vs. 103.9 months, Log rank P =0.0163) with similar survival to IPF (Log rank P =0.76). RA-UIP patients were older and predominantly male, though with similar baseline CT and PFT findings compared to other CTD-UIP (data not shown). Subgroup analysis of survival in UCTD-UIP (the most frequent CTD-UIP) was also done comparing survival to the other CTD-UIP (inclusive and exclusive or RA-UIP) without statistical difference (Log rank 0.144) while UCTD-UIP compared to IPF was better (Log rank <0.0010) (Kaplan-Meier curves not shown).

As patient reported duration of symptoms prior to diagnosis was no different between IPF and CTD-UIP, survival analysis from time of first respiratory symptoms did not improve survival in either cohort (data not shown). Analysis from initial clinical ILD diagnosis also did not attenuate differences in survival.

## Discussion

Our study represents the largest cohort to date of biopsy confirmed CTD-UIP patients, comparing baseline clinical features and predictors of diagnosis and survival to biopsy confirmed IPF. Over 600 consecutive patients were studied with the majority undergoing biopsy because of initial clinical equipoise with ILD diagnosis.

Our main findings support better survival in patients with CTD-UIP compared to IPF, despite the presence of similar initial radiologic and PFT findings. Frequency of positive autoimmune serologies was higher in CTD than IPF, though still occurred in 29% of tested IPF patients. Clinical predictors for survival in CTD-UIP were age and FVC, compared to IPF which included additionally gender and DLCO. Atypical or additional pathologic findings such as organizing pneumonia, lymphoid aggregates, or chronic inflammation were more frequently seen in CTD-UIP, but did not predict survival ((HR 0.83 (0.59-1.14), P =0.27)in either cohort. In patients with pathologically confirmed UIP, younger age, female gender, presence of positive autoimmune serology, and atypical radiologic findings increased the risk of underlying CTD diagnosis. Finally, despite CTD-UIP overall having better survival, RA-UIP had similar survival to IPF with worse survival compared to other CTD-UIP in our cohort.

UIP represents a commonly found interstitial pneumonia pattern whose defining features belie a heterogenously progressive fibrotic process characterized by fibroblast foci and honeycombing. Characteristic pathologic findings may be diagnosed variably among evaluating pathologists, though little is known about the prognostic features of atypical findings such as organizing pneumonia or lymphoid hyperplasia in the setting of histopathologic UIP pattern other than their association with CTD. Prior studies have suggested increased lymphoid aggregates [22] or hyperplasia [23] associated with underlying CTD, along with decreased number of fibroblast foci [23,24]. The specific features of pathologic findings in UIP have also been predictive of survival in IPF, in particular the frequency of fibroblast foci [25]. The study by Park et al. compared pathologic features among differing ILD groups and noted improved survival among those with CTD-UIP compared to IPF [2]. Others have found no difference in survival among those with IPF and general CTD-ILD [12] or with autoimmune dominant lung fibrosis [26] not fitting CTD criteria. Little is known about the pathologic mechanisms that lead to a common final pathway of UIP pattern yet different rates of disease progression and survival. An understanding of the perhaps presumptive inflammatory-based mechanisms that lead to UIP in CTD-ILD compared to the relatively unknown mechanisms in IPF may be helpful in deciphering future IPF treatment approaches.

Our study highlights several insights in comparing biopsy confirmed UIP in IPF and CTD. As most patients with CTD-ILD have better prognosis than IPF, confirmatory biopsy appears unnecessary to justify treatment as response to therapy appears better among all pathologic subclasses. Even so, most patients undergoing biopsy do so as a result of clinical equipoise in defining early or atypical lung fibrosis radiologically with possible or inconsistent IPF features and no clearly defined CTD or secondary cause. In our cohort, presenting radiologic and PFT findings were no different in those with suspected IPF and eventual CTD. Prior studies support the relatively high frequency of positive autoimmune serologies in IPF further confounding diagnostic evaluation in early disease [27,28]. Our study confirms that even among patients with UIP, female gender, younger age, atypical CT findings, and positive autoimmune serology were still predictive of subsequent CTD. On the other hand UIP pathology with inconsistent UIP CT pattern occurs relatively frequently in IPF with similar survival to radiologically consistent disease. A recent study proposes perhaps increased frequency of acute exacerbation in IPF compared to CTD-ILD as a reason for this difference in survival, as deterioration of PFT findings over time appeared similar between the two groups [29].

In our pathologically defined cohort, UIP associated with RA appears to have similar clinical presentation and survival to IPF. In particular, RA patients were older with more male predominance compared to other CTD-ILD, and did worse despite similar presenting PFT and CT patterns among all CTD patients. A recent study suggested better survival in comparison to case-matched IPF controls who received treatment [30]. While an NSIP CT pattern and histology are most common in CTD-ILD, recent work suggests UIP CT features may occur more frequently than previously noted [31] and is highly consistent with underlying UIP pathology [20]. Such radiologic features of advanced disease appear predictive of survival similar to IPF, though in our cohort, similar presenting radiologic patterns were seen between RA-UIP patients and other CTD-UIP (predominantly atypical or probable UIP CT findings). As UIP pathology appears to bode worse survival, confirmatory biopsy in this subset of CTD-ILD particularly with atypical CT may not be unreasonable. Underlying mechanisms as to why RA-UIP may do poorly is currently unknown and the subject of ongoing study.

Survival in IPF is known to be variable and may be affected by multiple factors including disease severity at the time of presentation and access to tertiary or expert care with earlier assessment [32]. Whether earlier diagnosis affects outcome remains tentative, as most patients with consistent UIP pattern on CT likely represent more advanced disease with worse survival. We note survival by 5 year intervals in our cohort suggest recently biopsied patients had better survival than those biopsied a little over a decade ago. This may be explained by perhaps earlier diagnosis and better recognition of disease, but unlikely secondary to improved treatments. One hypothesis is perhaps a trend away from empiric steroids and other anti-inflammatory agents in recent years for the treatment of IPF, which may have had previously deleterious effects on survival outside of disease progression itself. This is reflected in the comparatively worse survival of those on combination anti-inflammatory therapy with prednisone and azathioprine as found with the PANTHER trial [33].

Limitations of our study include its retrospective approach and the inclusion of only biopsy confirmed patients. As the intent of the study was to assess the role of underlying pathology and its implication in prognosis and response to treatment, application of our findings to clinically suspected IPF or CTD-ILD patients without confirmed biopsy may be limited. NSIP still remains the dominant pathologic finding in CTD-ILD where survival among non-biopsied patients may be more influenced by this than the noted improved survival of CTD-UIP. Our cohorts suggest that the presence of diagnosed CTD and not UIP itself appears to portend better survival even among patients with initially similar pathology, radiology, and PFT findings. Finally, while CTD-UIP other than RA-UIP had better survival than IPF, seven rheumatologic diseases were represented with heterogenous and unique clinical courses, whose survival or response to therapy may be marked by other variables outside of lung fibrosis.

In summary, our study is the largest to date comparing survival in IPF vs CTD-UIP, noting clinical predictors of disease progression with improved survival among those with CTD-UIP except RA-UIP which had similar survival to IPF. Clinical manifestations among those with unspecified UIP that may predict CTD include female gender, younger age, inconsistent radiologic findings, and positive autoimmune serology. Radiologic and pathologic findings alone and corrected were not predictive of either CTD-ILD diagnosis or prognosis.
